# Estimating Maximal Aerobic Speed in Academy Soccer Players: A Comparison Between Time Trial Methods and the 30‐15 Intermittent Fitness Test

**DOI:** 10.1002/ejsc.12315

**Published:** 2025-05-12

**Authors:** Kieran Smith, Matthew D. Wright, Paul Chesterton, Jonathan M. Taylor

**Affiliations:** ^1^ Middlesbrough FC Middlesbrough UK; ^2^ School of Health and Life Sciences Teesside University Middlesbrough UK

**Keywords:** aerobic fitness, field testing, MAS, physical performance, team sports

## Abstract

Aerobic fitness is crucial to team sport performance and can be practically estimated through field‐based assessments of maximal aerobic speed (MAS) in soccer players. However, method comparisons are underexplored. This study compared MAS estimation via a 6‐min distance trial (6minDT), 1800‐m time trial (1800mTT) and 30‐15 intermittent fitness test (30‐15IFT) and qualitative preferences in academy soccer players. Twenty‐six male players (age: 17.2 ± 0.6 years; stature: 178.4 ± 5.1 cm; body mass: 71.8 ± 7.7 kg) completed a 30‐15IFT, 6minDT and 1800mTT in a counterbalanced order. Total distance was measured via 10 Hz global positioning system units, with MAS defined as 87% of 30‐15IFT finishing speed (*v*IFT) and distance divided by time to completion for the 6minDT and 1800mTT. Pearson's correlations calculated associations between MAS estimates, and a robust repeated measures ANOVA estimated marginal mean differences between tests. A very large association was observed between performance in the 1800mTT and 6minDT (*r* = 0.72; 95% confidence interval, 0.46 to 0.86). Large associations were observed between the 30‐15IFT and 6minDT and 1800mTT (6minDT: *r* = 0.65; 0.35 to 0.83; 1800mTT: 0.66, 0.37 to 0.84), respectively. MAS estimated from the 30‐15IFT was significantly higher than the 6minDT (0.57, 0.50 to 0.65 m·s^−1^, *p* < 0.0001) and 1800mTT (0.45, 0.37 to 0.52 m·s^−1^, *p* < 0.0001). MAS estimated from the 6minDT was lower than the 1800mTT (−0.13, −0.21 to −0.05 m·s^−1^, *p* = 0.0002). In conclusion, although associations between 6minDT‐, 1800mTT‐ and 30‐15IFT‐derived MAS exist, all tests provide distinct estimations of MAS.

**Trial Registration:**

This study was pre‐registered on 11/03/2024. The preregistration for this study can be found on OSF registries at the following Registration DOI: https://doi.org/10.17605/OSF.IO/F3Z5U

**Summary**

- Key differences are observed in MAS estimation, associations between tests and the qualitative experiences of players undergoing different MAS assessments.
- Despite significant differences in between‐test MAS estimations and player preferences, the distance (6minDT) and time (1800mTT) trials employed in this study may be used interchangeably to estimate MAS without any meaningful impact on subsequent training prescriptions.
- Eighty‐seven percent of *v*IFT provides a higher estimation of MAS compared to time and distance trial methods; we propose that the percentage correspondence of *v*IFT to MAS may be closer to ∼80% as a means of estimating MAS from the 30‐15IFT.

## 1 Introduction

Soccer match play is characterised by intermittent bouts of high‐intensity activity, with players covering total distances of 10–13 km (Sarmento et al. 2014). Approximately 22%–24% of this distance is achieved at higher intensities above 15 km·h^−1^, resulting in an intricate interplay between the aerobic and anaerobic energy systems (Dolci et al. 2020). Aerobic fitness, therefore, plays a crucial role in supporting player recovery from high‐intensity actions (Altmann et al. 2020). High‐intensity interval training (HIIT) is regularly employed to effectively improve cardiorespiratory and metabolic function and, in turn, physical performance (Buchheit and Laursen 2013). However, appropriate assessment of player physical profiles has the potential to enhance the precision and accuracy of training prescriptions, leading to improved physiological adaptation and lowered risk of nonresponse or overreaching (Baker and Heaney 2015; Malone et al. 2017). In the context of academy players, who are in a critical developmental phase, improving the accuracy of these prescriptions could optimise performance by better aligning training stimuli with each player's capacity for work (Buchheit and Laursen 2013; Tears et al. 2018; Taylor et al. 2022).

Maximal aerobic speed (MAS), defined as the lowest running velocity at which maximal oxygen uptake (*V̇*O_2max_) is achieved, serves as a foundational measure of aerobic fitness, which is critical for sustained performance in sports with high‐intensity intermittent running demands, such as soccer (Buchheit and Laursen 2013). With this relationship also shown to intensify with age, MAS is reported to correlate with high‐intensity intermittent running ability across male players at U14 (*r* = 0.67), U16 (*r* = 0.73) and U18 (*r* = 0.87) (Buchheit and Mendez‐Villanueva 2013). Thus, MAS appears to be a valid measure of a player’s capacity to sustain high‐intensity intermittent running demands and, consequently, has also become a well‐established metric for prescribing HIIT interval intensities (Buchheit and Laursen 2013). Combined with maximal sprinting speed, MAS can also be used to calculate the anaerobic speed reserve (ASR), which represents the difference between a player's maximal aerobic capacity and maximal sprinting speed (MSS) (Sandford et al. 2021). This has the potential to provide further insight into an athlete's capacity for supramaximal efforts (Sandford et al. 2021).

Field‐based fitness assessments offer valuable insights into players' physical profiles within an ecologically valid environment (Altmann et al. 2020; Thron et al. 2024). Unlike laboratory‐based tests, which often require expensive, sophisticated equipment and an increased time burden, field‐based tests are more accessible and cost‐effective and provide a practical means to quantify physical performance attributes such as aerobic fitness (through estimated maximal aerobic speed) (Baker and Heaney 2015). Although laboratory‐based tests, though precise, may accurately determine an athlete's physiological capacity, it is suggested that field‐based tests (particularly intermittent protocols) may more closely replicate how HIIT is prescribed in most intermittent team sports (Buchheit and Laursen 2013). This often leads to a preference for intermittent protocols in team sports (Baker and Heaney 2015; Asimakidis et al. 2024).

Although laboratory‐based cardiopulmonary exercise testing (CPET) using a graded exercise test with direct measurement of expired air remains the gold standard for assessment of aerobic fitness, various field‐based tests are used to estimate MAS, including time trials running for distance lasting 5–7 min in duration and time trials running for time over courses ranging from 1000 to 2200 m (Asimakidis et al. 2024). With regards to intermittent tests, the 30‐15 intermittent fitness test (30‐15IFT) has become a popular means of assessing aerobic fitness due to its utility in HIIT prescription (Buchheit 2010; Asimakidis et al. 2024). Time and distance trials provide a straightforward estimate of MAS but require players to be familiar with appropriate pacing strategies (Thron et al. 2024). The 30‐15IFT may simulate the intermittent nature of soccer match play more effectively than time trials but does not specifically aim to estimate MAS, and this is often a secondary consideration when using this test, with greater value offered around supramaximal exercise capacity proposed (Buchheit 2010). Given the time constraints and logistical challenges around fitness testing, practitioners are often tasked with choosing one method of aerobic assessment as part of their testing batteries (Weakley et al. 2024). Therefore, further understanding of the differences in MAS estimation via continuous time trials and intermittent protocols is needed to establish the ‘interchangeability’ of these tests and understand protocol‐dependent differences, which may also influence prescription optimisation.

Psychological factors such as perceived effort and enjoyment may also impact fitness test outcomes/effort; consequently, evaluating players' qualitative preferences of testing protocols might present valuable insight (Asimakidis et al. 2024). Given clear differences in common test formats used to estimate MAS and subsequent differences in what success looks such as in each test (i.e., achieving the highest distance or final speed or covering a given distance in the shortest time possible), awareness of player perceptions may help achieve greater athlete buy‐in to testing protocols, allowing practitioners to gain a true understanding of players’ fitness in maximal testing protocols such as aerobic fitness assessment. Therefore, understanding relative differences in estimated MAS from different testing methods and identifying players’ preferences may enable practitioners to make more informed choices.

Therefore, the primary aim of this study was to quantify the differences in MAS estimation between three testing protocols: (1) a 6‐min distance trial (6minDT); (2) an 1800‐m time trial (1800mTT) and (3) the 30‐15 intermittent fitness test (30‐15IFT; calculated as 87% of finishing speed) in academy soccer players. A secondary aim was to assess player preferences for these tests.

## 2 Methods

### 2.1 Study Design

An observational mixed‐methods design was used (Batterham and Hopkins 2006; Smith 2010) to compare estimated MAS and player qualitative preferences across three testing sessions. Before the commencement of this study, ethics clearance was received from Teesside University School of Health and Life Sciences (SHLS) research ethics subcommittee (Project ID: 19297).

### 2.2 Participants

Twenty‐six male academy soccer players (age: 17.2 ± 0.6 [15.5–18.5] years; stature: 178.4 ± 5.1 [170.0–193.2] cm; body mass: 71.8 ± 7.7 [60.0–90.4] kg) were recruited from two clubs in England, competing in the Under‐18 Premier League and National League Alliance Academy North, respectively. Following the participant classification framework established by McKay et al. (2022) for defining athletes’ training and performance calibre, the sample comprised Tier 3 (highly trained/national level) athletes. All participants were required to provide informed consent or parental consent and athlete assent were sought for players under the age of 18, prior to study commencement. Any player suffering from injury or a health condition that restricted their involvement in training or competition at the time of testing was excluded.

### 2.3 Protocols

**FIGURE 1:**
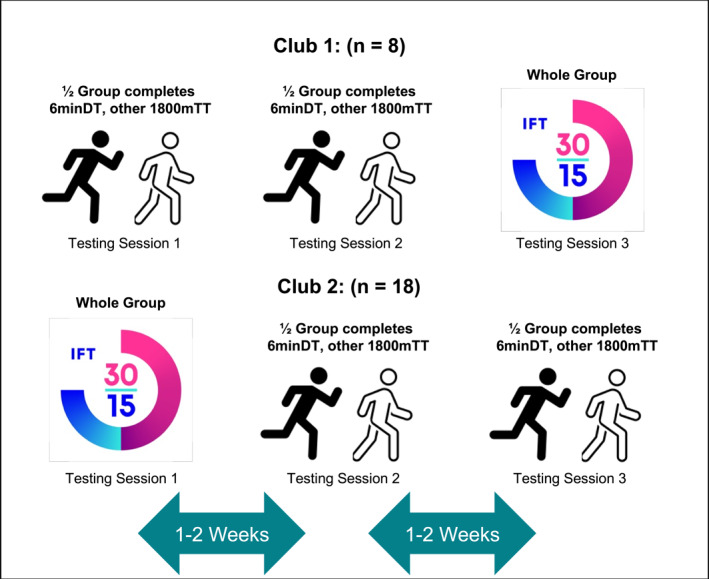
Schematic of study design. 6minDT: 6‐min distance trial; 30‐15IFT: 30‐15 intermittent fitness test; 1800mTT: 1800‐m time trial; n: number.

Testing took place over three separate sessions per club. Within the training microcycle, testing occurred 2 days after a match (MD + 2) or 3 days after (MD + 3), with each session separated by one to 2 weeks (Figure 1).

Players completed a standardised warm‐up prior to each test following the RAMP protocol (raise, activate, mobilise, potentiate [RAMP]) (Jeffreys 2006). The protocol comprised mobility and dynamic exercises before concluding with short accelerations to ensure players were physically and psychologically prepared for assessments and to reduce injury risk (Bishop 2003). Players completed all tests in a randomised, counterbalanced order at their respective clubs’ training facilities. Club 1 completed all testing on an artificial turf pitch in the following order: Week 1: 6minDT or 1800mTT; Week 2: 6minDT or 1800mTT; Week 3: 30‐15IFT. Club 2 completed testing in the reverse order of Club 1, with the 30‐15IFT assessment completed on an artificial turf pitch and the remaining time and distance trials completed on a full‐size grass pitch. All testing was completed under consistent environmental conditions (temperature: 20 ± 5.8°C; humidity: 65 ± 3.3%; pressure: 1010.2 ± 3.4 mbar; wind speed: 2.7 ± 0.4 km·h^−1^).

Total distance for the 6‐min distance trial (6minDT) and 1800‐m time trial (1800mTT) was measured using 10 Hz global positioning system units (OptimEye S5 and Vector S7, Catapult Innovations Pty Ltd, Australia) and software (Catapult OpenField Console v. 3.9, Catapult Innovations Pty Ltd, Australia). Assessment of total distance using OptimEye S5 and Vector S7 GPS units has previously been reported to demonstrate good convergent validity and excellent reliability at 10 Hz (CV: < 5%; ICC: > 0.9) (Akyildiz et al. 2022; Makar et al. 2023), with no difference in data filtering methods for calculating total distance between generations of units (Catapult Sports, 2023). Heart rate was monitored throughout the time and distance trials using chest‐worn heart rate monitors (Polar T31 and H10, Polar Electro Oy, Kempele, Finland). The 30‐15IFT assessments used in this study formed part of a larger battery of tests already being completed at respective clubs; therefore, HR data could not be collected for this test due to logistical constraints.

#### 2.3.1 Time and Distance Trials

**FIGURE 2:**
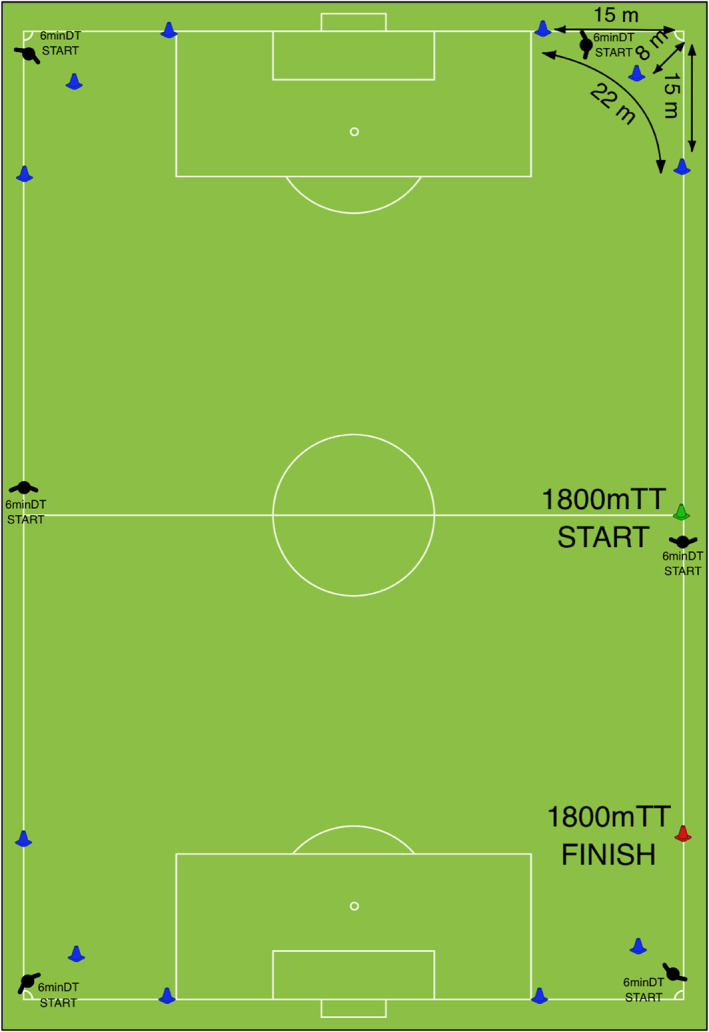
Custom track design for time and distance trials. 6minDT: 6‐min distance trial; 1800mTT: 1800‐m time trial; m: metre.

A 6‐min duration was selected due to being the typical time limit that MAS can be maintained during maximal effort aerobic running (Baker and Heaney 2015), and an 1800‐m distance was chosen given previously reported agreement with MAS derived from a graded incremental test (*r* = 0.81) (Bellenger et al. 2015). The 6minDT and 1800mTT were completed on a customised track (Figure 2).

Before the 6minDT, players were allocated to one of six starting points to evenly distribute players around the track geography (i.e., starting at a corner of the pitch or either side of the halfway line) (Figure 2); players started the test simultaneously from their assigned start position. Players were then instructed to cover as much distance as possible in the 6‐min period. The same custom course design was used for the 1800mTT, with all players starting from the nearside touchline at the halfway line (Figure 2), in small groups, staggered 15 s apart, to enable the test administrators to accurately time each player. The test was recorded using a video timer mobile application, ‘StopwatchCamera’ v. 1.2 (30 fps, 1080p) (newforestar co ltd 2024), to ascertain accurate finish times. Finish times for the 1800mTT were defined as the time in seconds, upon completion of the required number of laps (5 × full laps of the course + 1 × partial lap), when a player's foot crossed the finish line of the test. Intervals were called every 30 s to assist players with pacing during both tests. Physical performance and coaching staff closely monitored and verbally encouraged all players throughout both tests. Maximal aerobic speed (MAS) was calculated as the mean speed for the test (test distance [m]/time [s] = MAS m·s^−1^).

#### 2.3.2 30‐15 Intermittent Fitness Test (30‐15IFT)

The 30‐15IFT was delivered following the methods of Buchheit (2010) over a 40‐m track consisting of 30‐s shuttle runs interspersed with 15‐s passive recovery periods denoted by prerecorded instructions via the ‘30‐15 IFT’ mobile application v. 1.4 (Myorobie Buchheit 2024). Players continued running until either (a) volitional exhaustion or (b) they failed twice consecutively to reach a line before the audible cue. The last completed running speed (in km·h^−1^) was designated as *v*IFT (velocity for the intermittent fitness test). The test was also video recorded (30 fps, 1080p) (newforestar co ltd 2024) to ascertain accurate *v*IFT scores. To estimate MAS from the 30‐15IFT, Buchheit and Laursen (2013) have previously suggested a correction factor of 85%–90% of *v*IFT. This study, therefore, estimated MAS from the 30‐15IFT as 87% of the test final stage velocity (*v*IFT) (i.e., midpoint of the suggested percentage range).

#### 2.3.3 Qualitative Preferences of MAS Assessment Methods

Asimakidis et al. (2024) recently identified a lack of information regarding the specific physical performance testing preferences of key stakeholders (i.e., players, coaches and support staff) in elite soccer. Furthermore, careful consideration of how and what physical performance tests practitioners choose to implement should include both athlete buy‐in and familiarity with testing protocols, allowing for greater efficiency of testing procedures and their effectiveness in practice (Weakley et al. 2024). After completion of field‐based testing, player qualitative perspectives were collected. Questionnaire content was developed to relate to player perspectives regarding the assessment methods used, comprising familiarity with protocols, difficulty in pacing their effort during the test, motivation to complete the test and perceived usefulness of the test.

All qualitative data were collected in a controlled environment via a bespoke online form (Microsoft Forms, Microsoft Office 365 A3 2024, Microsoft Corp., Redmond, USA) (Appendix 1). Players recorded their answers using their own mobile devices, with the software uploading their responses to a cloud‐based spreadsheet (Microsoft Excel, Microsoft Office 365 A3 2024, Microsoft Corp., Redmond, USA). Players completed the form individually, without peer input, to reduce the likelihood of conscious reporting bias resulting from cognitive factors, such as misconception or comprehension of questions, or situational factors, such as deliberate deception in response to environmental, social or personal stimuli consistent with the team sport environment (McLaren et al. 2022). The estimated silent reading time of the form was calculated as 1 min and 2 s (245 [words on qualitative preferences form]/238 [average number of words per min when silent reading in English]) (Brysbaert 2019). The average response time to all six questions on the form was 3 min and 17 s ± 1 min and 12 s (range: 1 min and 27 s–5 min and 30 s) and used as an estimative measure of time on task (e.g., comprehension and active engagement with the questions).

### 2.4 Statistical Analysis

Data were collated and formatted using Microsoft Excel and analysed using R v. 4.4.3 (R Core Team 2025), the RStudio integrated development environment (IDE) v. 2024.12.1.563 (Posit team 2025) and relevant R packages (Appendix 3). Residuals were checked for violations of normality. Violations of normality were not present (Appendix 2). Consequently, descriptive statistics were utilised and presented as mean ± standard deviations (SD). The associations between MAS estimates were calculated using Pearson’s product–moment correlation coefficients. To limit the influence of any potential outliers (Field and Wilcox 2017), a robust repeated measures ANOVA was performed using the ‘robustlmm’ package v. 3.3.1 (Koller 2016). The ‘emmeans’ package v. 1.11.0 was then used to estimate marginal mean differences between tests (Lenth 2025). Correlation coefficients (*r*) were interpreted using the usual scale of thresholds: 0.1, small; 0.3, moderate; 0.5, large; 0.7, very large and 0.9, extremely large (Hopkins et al. 2009). Uncertainty in estimates was expressed as 95% confidence intervals (95%CI) for all analyses. Exact *p*‐values are also reported (to two significant digits for *p* ≥ 0.10 and one for *p* < 0.10) for the repeated measures ANOVA to express the probability of observing an effect as large or larger under the background statistical assumptions, including the null hypothesis, where true differences between tests were zero (Hopkins et al. 2009; Greenland et al. 2016). Statistical significance was set at *p* ≤ 0.05 or 5% (Batterham and Hopkins 2006; Hopkins et al. 2009). Example R code and all packages used in the statistical analysis of this study can be viewed here: https://rpubs.com/kieransmith/mas_comparison.

Data from multiple‐choice questions were converted into proportions with lower and upper limits of the 95% confidence interval calculated using the Wilson procedure (Newcombe 1998). With confidence intervals representing the uncertainty around the estimates (Greenland et al. 2016). Thematic analysis was adopted to examine responses to open‐ended questions, following the approach outlined by Braun and Clarke (2019). Two researchers (KS and PC) independently developed an initial coding framework, organising brief one‐ or two‐sentence responses within an Excel spreadsheet into thematic categories. As the analysis progressed, certain categories were merged, enabling a dynamic and iterative refinement of the coding structure with each new response. In the event of any disagreement regarding theme formation or coding, a third researcher (JT) would have conducted an independent review. A hermeneutic revisiting of the dataset minimised researcher bias, ensuring a thorough and balanced approach in identifying and developing categories.

## 3 Results

**FIGURE 3:**
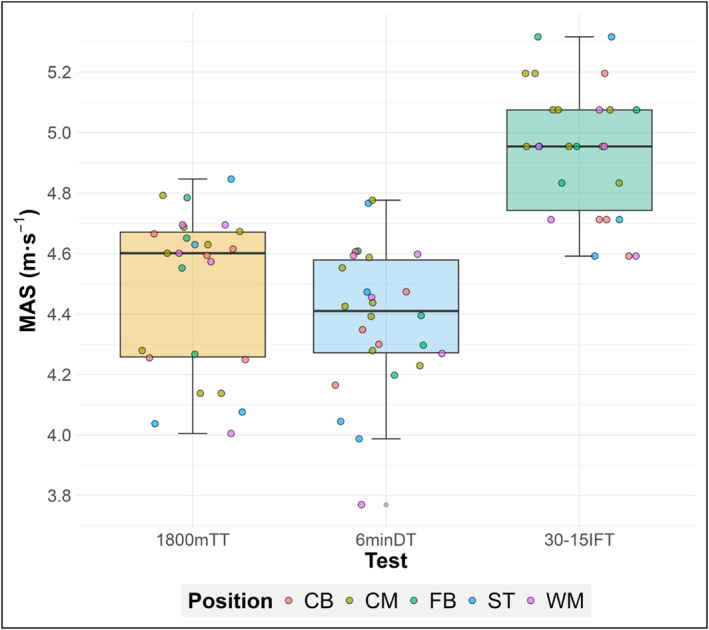
Box and whisker plot of distribution of 1800mTT‐, 6minDT‐ and 30‐15IFT‐derived MAS. 6minDT: 6‐min distance trial; 30‐15IFT: 30‐15 intermittent fitness test; 1800mTT: 1800‐m time trial; CB: centre back; CM: central midfielder; FB: fullback; MAS: maximum aerobic speed; m·s^−1^: metres per second; ST: striker; WM: wide midfielder.

Descriptive statistics for all tests are provided in Table 1, with MAS (m·s^−1^) from each test visualised in Figure 3. MAS estimated from the 30‐15IFT was significantly higher than that estimated from the 6minDT (0.57, 0.50 to 0.65 m·s^−1^, *p* < 0.0001) and 1800mTT (0.45, 0.37 to 0.52 m·s^−1^, *p* < 0.0001). The 6minDT resulted in a lower estimation of MAS compared to the 1800mTT (−0.13, −0.21 to −0.05 m·s^−1^, *p* = 0.0002). Differences between the 6minDT and 1800mTT were not significant for HR_avg_ (−4, −9 to 1 b·min^−1^, *p* = 0.24) and HR_peak_ (−3, −8 to 2 b·min^−1^, *p* = 0.42). However, large confidence intervals were observed and may have been negatively influenced by only 18 players from the overall sample having usable heart rate traces from both tests.

**TABLE 1:** Descriptive statistics (mean ± standard deviation, min–max) for field‐based testing outcomes.

Variable	Mean ± SD	Range (min–max)
6minDT		
MAS (m·s^−1^)	4.39 ± 0.24	3.77–4.78
Total distance (m)	1578.9 ± 84.6	1357.18–1719.62
HR_avg_ (b·min^−1^)	176 ± 10	160–192
HR_peak_ (b·min^−1^)	192 ± 11	163–207
1800mTT		
MAS (m·s^−1^)	4.49 ± 0.26	4.01–4.85
Finish time (s)	402.25 ± 24.15	371.4–449.4
HR_avg_ (b·min^−1^)	178 ± 14	136–195
HR_peak_ (b·min^−1^)	192 ± 15	136–203
30‐15IFT		
MAS (m·s^−1^)	4.94 ± 0.22	4.59–5.32
*v*IFT (km·h^−1^)	20.46 ± 0.89	19.00–22.00

Abbreviations: 6minDT: 6‐min distance trial; 30‐15IFT: 30‐15 intermittent fitness test; 1800mTT: 1800‐m time trial; b·min^−1^: beats per minute; HR_avg_: average heart rate; HR_peak_: peak heart rate; km·h^−1^: kilometres per hour; m: metres; MAS: maximum aerobic speed; Max: maximum; Min: minimum; m·s^−1^: metres per second; s: seconds; SD: standard deviation; *v*IFT: velocity for the 30‐15 intermittent fitness test.

**FIGURE 4:**
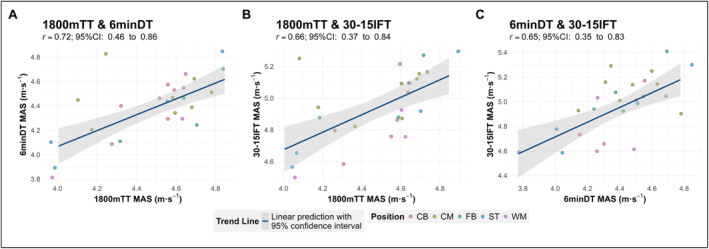
Linear models of relationships between 1800mTT‐, 6minDT‐ and 30‐15IFT‐derived MAS. (A) Scatter plot of 1800mTT‐ and 6minDT‐derived MAS. (B) Scatter plot of 1800mTT‐ and 30‐15IFT‐derived MAS. (C) Scatter plot of 6minDT‐ and 30‐15IFT‐derived MAS. 6minDT: 6‐min distance trial; 30‐15IFT: 30‐15 intermittent fitness test; 95%CI: 95% confidence interval; 1800mTT: 1800‐m time trial; CB: centre back; CM: central midfielder; FB: fullback; MAS: maximum aerobic speed; m·s^−1^: metres per second; *r*: correlation coefficient; ST: striker; WM: wide midfielder.

All associations between tests were positive. Associations between the 1800mTT and 6minDT ranged from moderate to very large (*r* = 0.72; 95% confidence interval, 0.46 to 0.86, Figure 4A). Moderate to very large associations were also observed between the 30‐15IFT and the 6minDT (*r* = 0.65; 0.35 to 0.83) and 1800mTT (0.66, 0.37 to 0.84) (Figure 4B,C).

### 3.1 Qualitative Preferences

Table 2 shows respondents’ preference of test and subsequent reasoning relating to familiarity, pacing, regulation of effort, challenge, motivation and perception of how well the test represented their ability to work aerobically.

**TABLE 2:** Player qualitative preference of test.

Multiple‐choice questions	18 respondent answers		
	*n* (%, 95%CI)		
	6minDT	1800mTT	30‐15IFT
Which test did you prefer?	13 (72, 49 to 88)	3 (17, 6 to 39)	2 (11, 3 to 33)
a. I was more familiar with this test protocol.	8 (44, 25 to 66)	3 (17, 6 to 39)	7 (39, 20 to 61)
b. I found the audible cues used in this test (e.g., time called every 30 s or beeps) easier to pace my effort in relation to the end of this test.	4 (22, 9 to 45)	7 (39, 20 to 61)	7 (39, 20 to 61)
c. The endpoint of this test was easier to interpret in relation to my effort throughout the test.	4 (22, 9 to 45)	11 (61, 39 to 80)	3 (17, 6 to 39)
d. If I were to repeat this test using the same protocols, I would know how to adjust my pacing strategy to attempt to improve upon my previous result.	8 (44, 25 to 66)	5 (28, 13 to 51)	5 (28, 13 to 51)
e. I felt more motivated to complete this test.	5 (28, 13 to 51)	3 (17, 6 to 39)	10 (56, 34 to 75)
f. I feel this test is more representative of my ability to work aerobically.	7 (39, 20 to 61)	5 (28, 13 to 51)	6 (33, 16 to 56)

Abbreviations: 6minDT: 6‐min distance trial; 30‐15IFT: 30‐15 intermittent fitness test; 95%CI: 95% confidence interval; 1800mTT: 1800‐m time trial; n: number; %: percent.

Respondents were asked to explain why they felt more motivated to complete their preferred test. From open‐ended questioning, six themes emerged: running for distance, challenge, achieving a high score, format of the test, position‐specific/game replication and pacing. Concerning running for distance, respondents frequently cited knowing the endpoint of the test as valuable to their motivation to complete the test, whereas frequent mention of self‐competition was attributed to how well the test challenged each participant. Concerning the challenge aspect of the tests, achieving a high score was important to respondents. Respondents were asked to explain why they believed this test better represented their ability to work aerobically. This often depended on participants’ preference for test format (e.g., intermittent or continuous protocols). Similarly, how well each test provided position‐specific/game activity replication positively contributed to some players’ preference for the test (e.g., favouring the stop‐start nature of intermittent protocols over continuous formats) and feelings that this better represented their ability to work aerobically. This was also considered an important element of pacing, whereby players frequently mentioned the longer duration of the 1800mTT compared to the 6minDT, requiring more effort to pace effectively. However, the 6minDT was favoured by multiple respondents as a better representation of their ability to work aerobically due to their capacity to maintain a similar pace throughout the entire test.

## 4 Discussion

This study compared MAS estimation using a 6‐min distance trial (6minDT), 1800‐m time trial (1800mTT) and the 30‐15 intermittent fitness test (30‐15IFT) in academy soccer players. A secondary aim was to qualitatively evaluate player preferences between these tests. Our data indicated that MAS estimated as 87% of 30‐15IFT *v*IFT was significantly higher than that estimated from the 6minDT and 1800mTT. The 6minDT resulted in the lowest estimation of MAS compared to the 1800mTT and 30‐15IFT, with moderate to very large associations between the 1800mTT and 6minDT and moderate to very large associations observed between the 30‐15IFT and 6minDT and 1800mTT. These findings provide implications for the utility of different MAS assessment methods and highlight that although associations exist between MAS estimated from the 6minDT, 1800mTT and 30‐15IFT, all three tests provide different estimations of MAS. Most notably, 87% of 30‐15IFT *v*IFT, as a method for estimating MAS, provides a higher estimate of MAS and may lead to potentially problematic HIIT prescriptions with imprecise interval intensities, where the intention is to anchor to MAS. Secondly, although 6minDT‐ and 1800mTT‐derived MAS are significantly different from each other, both provide a similar physiological response with little meaningful effect on HIIT prescription. Further implications for test selection relating to players’ preference for testing protocols were also identified, with an overall player preference for the 6minDT to estimate MAS.

The observation that 30‐15IFT *v*IFT yields higher final speeds and subsequently higher MAS scores when estimated as 87% of *v*IFT compared to the average speed across 6minDT and 1800mTT underpins the findings of Buchheit (2008), reporting intermittent tests to allow for higher peak speeds due to greater anaerobic contributions. Continuous format tests such as the 6minDT and 1800mTT emphasise sustained aerobic capacity without recovery periods, which may lead to lower, potentially more accurate estimations of MAS (Baker and Heaney 2015). However, as previously mentioned, our data revealed that 87% of *v*IFT provides a higher estimate of MAS, leaving large unexplained variance when compared to time or distance trials. Consequently, when calculating percentage correspondence using the raw ratio of the observed data (percentage correspondence = [6minDT km·h^−1^ or 1800mTT km·h^−1^/*v*IFT km·h^−1^] × 100), our data suggest more realistic percentages of 77 ± 3% to obtain 6minDT MAS and 79 ± 3% to obtain 1800mTT MAS, suggesting MAS percentage correspondence to *v*IFT may be closer to ∼80%, challenging the previously reported percentage range of 85%–90% (Buchheit and Laursen 2013). However, these percentages should be used cautiously given the differences between test formats and the large confidence intervals observed for the associations between 1800mTT, 6minDT and 30‐15IFT. We also acknowledge that both time and distance trials may underestimate or overestimate MAS, just as an intermittent format test could. Specifically, this indicates that MAS estimates compare well between time or distance trials for some players but not for others. We propose that the following regression equations may be used to estimate MAS from *v*IFT km·h^−1^: 6minDT km·h^−1^ = 3.24 + 0.61 × *v*IFT km·h^−1^ (standard error: 0.69 km·h^−1^) and 1800mTT km·h^−1^ = −0.93 + 0.84 × *v*IFT km·h^−1^ (standard error: 0.37 km·h^−1^). However, given the high standard error, we acknowledge that further study is needed to validate these equations against gold‐standard laboratory‐based assessment.

Concerning practically important differences between tests and their subsequent effects on HIIT interval intensity prescription, the lower estimated MAS observed in the 6minDT compared to the 1800mTT may reflect differences in pacing strategies, as pacing is reported to be enhanced when the test endpoint is defined by distance rather than time (Duffield et al. 2005; Jones et al. 2015). However, given that the smallest worthwhile change in MAS is proposed to be 0.2 m·s^−1^ (Thron et al. 2024), our data suggest practical equivalence whereby while still acknowledging error in the prediction, time and distance trials may be used interchangeably, with the observed difference in our study being < 0.2 m·s^−1^ (Lakens 2017). This is further compounded given the little physiological difference between the time and distance trial protocols used, exhibiting comparable HR indices (HR_avg_ and HR_peak_ b·min^−1^). In context, our data suggest negligible difference would be observed in HIIT prescription depending on the time and distance protocols we used. For example, a typical short interval HIIT session (e.g., work:rest: 1:2; @115% MAS; 2 × [6 × 20 s]; 40 s rest between reps; 3 min between sets) would result in a one‐repetition total distance of 101 m when prescribed using 6minDT MAS and 103 m when prescribed using 1800mTT MAS (i.e., a difference of 24 m in session total distance between prescriptions). By comparison, prescription using 87% of 30‐15IFT *v*IFT would result in a 114‐m one‐repetition total distance (or ∼140 m across the entire session).

The associations identified between tests suggest that although they assess similar components of aerobic fitness, each test emphasises different physiological characteristics (i.e., the distance/time trials vs. 30‐15IFT). The association between the continuous format tests (6minDT and 1800mTT) was expected due to their reliance on sustained energy delivery via aerobic metabolism (Midgley et al. 2006). Although the association observed between the 30‐15IFT and the continuous tests reflects the test’s shared contributions from aerobic capacity, a likely greater anaerobic capacity is necessitated by frequent deceleration, change of direction and reacceleration over an extended period within intermittent format tests (Buchheit 2008). This dual emphasis of the test aligns with the movement patterns of soccer match play, where players must recover quickly between repeated high‐intensity efforts (Iaia et al. 2009). Consequently, where practitioners seek to estimate MAS for HIIT prescription, we recommend that practitioners either choose a time/distance trial method or utilise the lower percentage of 30‐15IFT *v*IFT (i.e., ∼80%) identified in this study to estimate MAS as well as *v*IFT. The 30‐15IFT appears to be more suited to supramaximal HIIT prescriptions, but practitioners may alternatively use estimated MAS from the distance/time trials in combination with MSS to calculate ASR to determine player physical profile type and prescribe supramaximal intervals (Buchheit et al. 2021; Sandford et al. 2021).

Player preference for the 6minDT, reported by 72% of respondents, highlights the psychological roles of familiarity, simplicity and pacing ease in aerobic fitness testing practices. Previous research has demonstrated that athletes are more motivated and perform better when they understand test structure and pacing requirements (Smits et al. 2014). Conversely, the 30‐15IFT was preferred by players who appreciated the challenging nature of this test and perceived alignment with game demands. This preference reflects the ecological validity of the test (i.e., similarity to the stop‐start nature of soccer match play) (Buchheit 2008). The motivational aspects of knowing the endpoint and self‐competition were frequently cited in qualitative responses, further emphasising the importance of psychological factors in test selection. However, it is acknowledged that these are the preferences of a very small sample of players and, although insightful, these responses will be biased by previous experiences of these testing methods. Furthermore, although most players appeared to spend an appropriate amount of time on task when providing qualitative preferences, given the reported response times to the form, many of the free‐text responses analysed lacked sufficient detail to draw any comprehensive conclusions.

### 4.1 Limitations

Although this study presents novel information, it is not without limitations. Firstly, we acknowledge that the sample size is relatively small and may have contributed to uncertainty in the precision of our estimates (Halperin et al. 2018). We also acknowledge the change in surface between the 30‐15IFT and time/distance trials for the second sample collected with Club 2. However, this is representative of ecological validity, where the changeable nature of team sport environments may necessitate schedule changes despite best efforts to maintain control over elements of study design (Weakley et al. 2024). Although testing took place in the summer months, the surface of the grass pitches was dry and firm due to consistent weather. Finally, HR data were only usable from 18 participants due to measurement error from equipment failure; therefore, HR data could only be included for a participant if present for both distance and time trials. Additionally, the 30‐15IFT assessments used in this study formed part of a larger battery of tests already being completed at clubs. HR data could, therefore, not be collected for this test due to logistical constraints.

### 4.2 Practical Applications

This study highlights key differences in MAS estimation, associations between tests and the qualitative experiences of players undergoing different MAS assessments. Despite significant differences in estimated MAS and player preference for the 6minDT, the distance (6minDT) and time (1800mTT) trials used here may be used interchangeably to estimate MAS without any meaningful impact on subsequent training prescriptions. Given the higher estimated MAS for the 30‐15IFT when calculated as 87% of *v*IFT, we propose that the percentage correspondence of *v*IFT to MAS may be closer to ∼80% as a means of estimating MAS from the 30‐15IFT. Future studies might consider differences and/or similarities in pacing strategies between time and distance trials, as well as intermittent format tests, to further understand their use among soccer players.

## 5 Conclusion

In conclusion, by carefully selecting a single aerobic fitness assessment or combining multiple tests while incorporating player feedback, practitioners may obtain a more comprehensive evaluation of player fitness and better tailor training interventions to player needs. Specifically, careful selection of testing protocols is crucial to the appropriate estimation of MAS, although using a combination of tests may also provide a more holistic understanding of players' fitness and better inform individualised training strategies.

## Author Contributions

Conceptualisation: Kieran Smith, Matthew D. Wright, Paul Chesterton and Jonathan M. Taylor. Data Curation: Kieran Smith, Matthew D. Wright, Paul Chesterton and Jonathan M. Taylor. Formal Analysis: Kieran Smith, Matthew D. Wright, Paul Chesterton and Jonathan M. Taylor. Investigation: Kieran Smith. Methodology: Kieran Smith, Matthew D. Wright, Paul Chesterton and Jonathan M. Taylor. Project Administration: Kieran Smith and Jonathan M. Taylor. Resources: Kieran Smith, Matthew D. Wright, Paul Chesterton and Jonathan M. Taylor. Visualisation: Kieran Smith, Matthew D. Wright, Paul Chesterton and Jonathan M. Taylor. Writing – Original Draft: Kieran Smith, Matthew D. Wright, Paul Chesterton and Jonathan M. Taylor. Writing – Review and Editing: Kieran Smith, Matthew D. Wright, Paul Chesterton and Jonathan M. Taylor.

## Ethics Statement

Ethical approval was obtained from Teesside University School of Health and Life Sciences (SHLS) research ethics subcommittee, Project ID: 19297.

## Consent

All participants gave informed consent to participate in this research.

## Conflicts of Interest

The authors declare no conflicts of interest.

## Code Availability Statement

The code used to analyse the data used in this study is available upon request or openly via OSF repository at: https://osf.io/8u6yj.

## Data Deposition

The data supporting this study's findings are openly available via OSF repository at: https://osf.io/gqutc.

## Supporting information

Supporting Information S1

## Data Availability

The data supporting this study's findings are available upon request or openly via the OSF repository here: https://osf.io/gqutc.
