# Supplementary material for: Estimating Maximal Aerobic Speed in Academy Soccer Players: A Comparison Between Time Trial Methods and the 30‐15 Intermittent Fitness Test
Source: Eur J Sport Sci. 2025 May 12;25(6):e12315. doi: 10.1002/ejsc.12315 (PMC12069019; doi:10.1002/ejsc.12315)
Supplement: Supplementary file 1 — Supporting Information S1 [file EJSC-25-e12315-s001.docx]

**Title:** Estimating Maximal Aerobic Speed in Academy Soccer Players: A Comparison Between Time-Trial Methods and the 30-15 Intermittent Fitness Test

# SUPPORTING INFORMATION

## Qualitative Preferences Questionnaire

Interchangeability of Time vs Distance trials vs 30-15 IFT in estimating MAS

1. Name
2. Reflecting on the 3 field-based tests you have completed; 6 min run (DT), 1800 m time trial (TT) and 30-15 IFT, which did you prefer?
   1. 6 min run (DT)
   2. 1800 m time trial (TT)
   3. 30-15 IFT
3. Reflecting on your experience of undertaking the 3 field-based tests you have completed; 6 min run (DT), 1800 m time trial (TT) and 30-15 IFT, rate each of the following statements against the 3 tests.
   1. I was more familiar with this test protocol.
   2. When times were called every 30 seconds, I found it easier to pace my effort in relation to the end of this test.
   3. The endpoint of this test was easier to interpret in relation to my effort throughout the test.
   4. If I were to repeat this test using the same protocols, I would know how to adjust my pacing strategy to attempt to improve upon my previous result.
   5. I felt more motivated to complete this test.
   6. I feel this test is more representative of my ability to work aerobically.
4. [FREE TEXT] Please explain your answer to statement e., why did you feel more motivated to complete this test?
5. [FREE TEXT] Please explain your answer to statement f., why did you feel this test is more representative of your ability to work aerobically?
6. [FREE TEXT] Are there any other reasons for your preference of test not already mentioned?

##
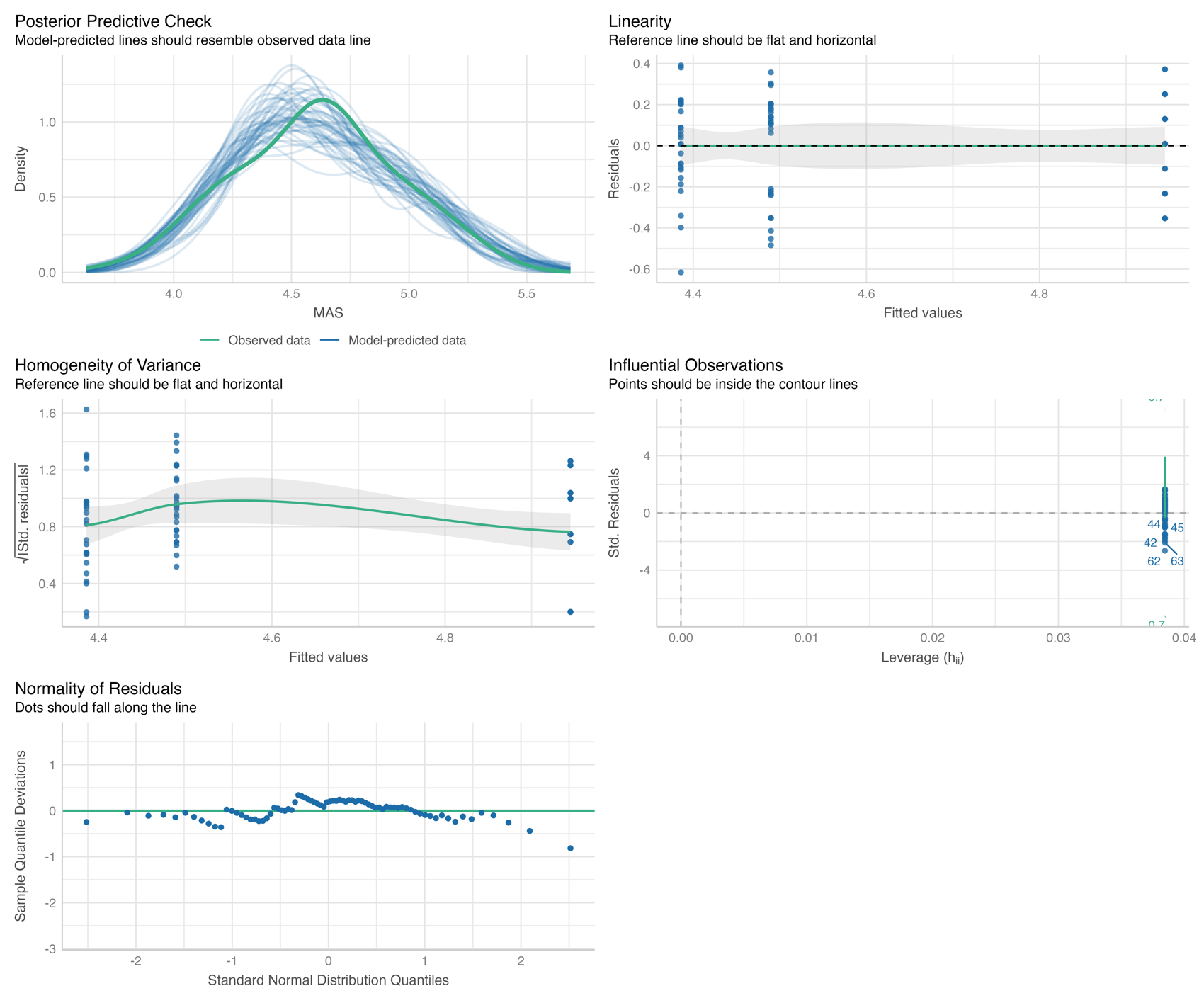
Model Normality

## Original Full Output Generated by RStudio

R version 4.4.3 (2025-02-28) -- "Trophy Case"

Copyright (C) 2025 The R Foundation for Statistical Computing

Platform: aarch64-apple-darwin20

R is free software and comes with ABSOLUTELY NO WARRANTY.

You are welcome to redistribute it under certain conditions.

Type 'license()' or 'licence()' for distribution details.

Natural language support but running in an English locale

R is a collaborative project with many contributors.

Type 'contributors()' for more information and

'citation()' on how to cite R or R packages in publications.

Type 'demo()' for some demos, 'help()' for on-line help, or

'help.start()' for an HTML browser interface to help.

Type 'q()' to quit R.

> # load packages ----------------------------------------------------------------

>

> library(readxl)

> library(janitor)

> library(tidyverse)

> library(writexl)

> library(patchwork)

> library(broom)

> library(performance)

> library(lme4)

> library(robustlmm)

> library(emmeans)

> library(robustbase)

> library(grateful)

> # set working directory --------------------------------------------------------

>

> setwd("~/Desktop")

> # load data & clean names ------------------------------------------------------

>

> # participant charcteristics & 1800mTT ----

> TT18 <- read .... [TRUNCATED]

-

/

/

-

> names <- names(TT18 %>% clean_names)

> colnames(TT18) <- names

> TT18 <-

+ TT18 %>% subset(

+ select = c(

+ player_id,

+ playing_level,

+ squad,

+ position,

+ ag .... [TRUNCATED]

> # 6minDT ----

> DT6 <- read_excel("study1data.xlsx", sheet = "6 min DT")

-

/

/

-

> names <- names(DT6 %>% clean_names)

> colnames(DT6) <- names

> DT6 <-

+ DT6 %>% subset(

+ select = c(

+ player_id,

+ x6_min_dt_total_distance_m,

+ mas_m_s,

+ avg_heart .... [TRUNCATED]

> # 30-15IFT ----

> IFT <- read_excel("study1data.xlsx", sheet = "30-15 IFT")

-

/

/

-

> names <- names(IFT %>% clean_names)

> colnames(IFT) <- names

> IFT <-

+ IFT %>% subset(

+ select = c(

+ player_id,

+ v_ift_km_hr,

+ mas_m_s,

+ temperature_c,

+ .... [TRUNCATED]

> # join 3 df's into single df "data" ----

> data <- TT18 %>%

+ full_join(DT6, by = "player_id") %>%

+ full_join(IFT, by = "player_id")

> # omit na values

> data <- data %>%

+ filter(!is.na(TT18_mas_m_s),

+ !is.na(DT6_mas_m_s),

+ !is.na(ift_mas_m_s))

> # view data df

> View(data)

> # data quick look summary stats

> summary(data)

player_id playing_level squad position age_years stature_cm body_mass_kg x1800_m_tt_finish_time_s TT18_mas_m_s TT18_avg_heart_rate_b_min TT18_peak_heart_rate_b_min

Length:26 Length:26 Length:26 Length:26 Min. :15.50 Min. :170.0 Min. :60.00 Min. :371.4 Min. :4.005 Min. :135.7 Min. :136.0

Class :character Class :character Class :character Class :character 1st Qu.:16.83 1st Qu.:175.2 1st Qu.:66.45 1st Qu.:385.4 1st Qu.:4.258 1st Qu.:175.9 1st Qu.:193.0

Mode :character Mode :character Mode :character Mode :character Median :17.25 Median :176.9 Median :71.00 Median :391.2 Median :4.601 Median :180.1 Median :195.0

Mean :17.15 Mean :178.4 Mean :71.85 Mean :402.3 Mean :4.490 Mean :177.7 Mean :191.8

3rd Qu.:17.47 3rd Qu.:181.9 3rd Qu.:76.28 3rd Qu.:422.7 3rd Qu.:4.671 3rd Qu.:185.4 3rd Qu.:195.8

Max. :18.54 Max. :193.2 Max. :90.40 Max. :449.4 Max. :4.847 Max. :194.5 Max. :203.0

NA's :8 NA's :8

temperature_c.x humidity_percent.x pressure_mbar.x wind_speed_mph.x x6_min_dt_total_distance_m DT6_mas_m_s DT6_avg_heart_rate_b_min DT6_peak_heart_rate_b_min temperature_c.y humidity_percent.y pressure_mbar.y

Min. :11.60 Min. :66.20 Min. : 996.6 Min. :1.000 Min. :1357 Min. :3.770 Min. :159.6 Min. :163.0 Min. :11.60 Min. :66.20 Min. : 996.6

1st Qu.:12.80 1st Qu.:66.20 1st Qu.:1007.8 1st Qu.:1.000 1st Qu.:1538 1st Qu.:4.272 1st Qu.:169.2 1st Qu.:189.0 1st Qu.:12.80 1st Qu.:66.20 1st Qu.:1007.8

Median :21.00 Median :71.90 Median :1009.5 Median :2.000 Median :1588 Median :4.410 Median :176.1 Median :193.0 Median :21.00 Median :71.90 Median :1009.5

Mean :19.52 Mean :70.45 Mean :1008.5 Mean :1.692 Mean :1579 Mean :4.386 Mean :176.0 Mean :191.8 Mean :19.83 Mean :70.88 Mean :1008.8

3rd Qu.:25.00 3rd Qu.:73.20 3rd Qu.:1013.5 3rd Qu.:2.000 3rd Qu.:1648 3rd Qu.:4.579 3rd Qu.:184.4 3rd Qu.:199.5 3rd Qu.:25.00 3rd Qu.:73.20 3rd Qu.:1013.5

Max. :25.00 Max. :75.40 Max. :1013.5 Max. :2.900 Max. :1720 Max. :4.777 Max. :192.3 Max. :207.0 Max. :25.00 Max. :75.40 Max. :1013.5

NA's :8 NA's :8

wind_speed_mph.y v_ift_km_hr ift_mas_m_s temperature_c humidity_percent pressure_mbar

Min. :1.000 Min. :19.00 Min. :4.592 Min. :10.00 Min. :50.00 Min. :1012

1st Qu.:1.000 1st Qu.:19.62 1st Qu.:4.743 1st Qu.:10.00 1st Qu.:50.00 1st Qu.:1012

Median :1.100 Median :20.50 Median :4.954 Median :26.00 Median :50.00 Median :1013

Mean :1.615 Mean :20.46 Mean :4.945 Mean :20.73 Mean :53.69 Mean :1013

3rd Qu.:2.000 3rd Qu.:21.00 3rd Qu.:5.075 3rd Qu.:26.00 3rd Qu.:58.00 3rd Qu.:1013

Max. :2.900 Max. :22.00 Max. :5.317 Max. :26.00 Max. :82.00 Max. :1028

> # participant characteristics --------------------------------------------------

>

> # overall summary (without squad grouping)

> overall_cha .... [TRUNCATED]

> # create squad level participant characteristics df

> parcharacteristics <- data %>%

+ select(squad, age_years, stature_cm, body_mass_kg) %> .... [TRUNCATED]

> # combine squad-level and overall-level summaries

> parcharacteristics <- bind_rows(overall_characteristics, parcharacteristics) %>%

+ # Rea .... [TRUNCATED]

> # view parcharacteristics df

> View(parcharacteristics)

> # export to .xlsx

> write_xlsx(list("Participant Characteristics" = parcharacteristics),

+ "Study 1 Results.xlsx")

> # descriptive stats ------------------------------------------------------------

>

> # count of player positions ----

> position_counts <- da .... [TRUNCATED]

> # view position_counts df

> view(position_counts)

> # export to .xlsx

> write_xlsx(

+ list(

+ "Participant Characteristics" = parcharacteristics,

+ "Position Counts" = position_cou .... [TRUNCATED]

> # conditions across testing sessions ----

> conditions <- data %>%

+ select(

+ player_id,

+ contains("temperature"),

+ con .... [TRUNCATED]

> # avg_cond df

> avg_cond <- conditions %>%

+ select(temperature_c,

+ humidity_percent,

+ pressure_mbar,

+ w .... [TRUNCATED]

> # view avg_cond df

> view(avg_cond)

> # export to .xlsx

> write_xlsx(

+ list(

+ "Participant Characteristics" = parcharacteristics,

+ "Position Counts" = position_cou .... [TRUNCATED]

> # descriptive stats df ----

> descriptivestats <-

+ data %>% # select data

+ selec .... [TRUNCATED]

> # view descriptivestats df

> View(descriptivestats)

> # export to .xlsx

> write_xlsx(

+ list(

+ "Participant Characteristics" = parcharacteristics,

+ "Position Counts" = position_cou .... [TRUNCATED]

> # MAS distribution -------------------------------------------------------------

>

> # organise data long ----

> data2 <- data %>%

+ sele .... [TRUNCATED]

> data_long <- pivot_longer(

+ data2,

+ cols = c(ift_mas_m_s, DT6_mas_m_s, TT18_mas_m_s),

+ names_to = "Test",

+ values_to = "MAS"

+ .... [TRUNCATED]

> # view data_long df

> View(data_long)

> # create plot theme

> plottheme <- theme(

+ plot.margin = margin(10, 10, 10, 10),

+ text = element_text(family = "Arial"),

+ plot.ti .... [TRUNCATED]

> # box and whisker plot ----

> # colourblind palette for boxplot

> cb_box_palette <- c(

+ 'TT18_mas_m_s' = "#E69F00",

+ 'DT6_mas_m_s' .... [TRUNCATED]

> # plot

> plot1 <-

+ ggplot(data_long, aes(x = factor(

+ Test, levels = c('TT18_mas_m_s', 'DT6_mas_m_s', 'ift_mas_m_s')

+ ), y = MA .... [TRUNCATED]

> # print plot

> plot1

> # export plot

> ggsave(

+ "Box Plot.png",

+ plot = plot1,

+ bg = 'white',

+ dpi = 600,

+ width = 9,

+ height = 8,

+ .... [TRUNCATED]

> # relationships & linear models ------------------------------------------------

>

> # pearson's product-moment correlation coefficients ----

> .... [TRUNCATED]

> # 1800mTT & 30-15IFT

> cor2 <-

+ cor.test(

+ data$TT18_mas_m_s,

+ data$ift_mas_m_s,

+ use = "pairwise.complete.obs",

+ .... [TRUNCATED]

> # 6minDT & 30-15IFT

> cor3 <-

+ cor.test(

+ data$DT6_mas_m_s,

+ data$ift_mas_m_s,

+ use = "pairwise.complete.obs",

+ .... [TRUNCATED]

> # extract statistics from each correlation test

> pcc1 <- data.frame(

+ Test = "1800mTT & 6minDT",

+ Estimate = cor1$estimate,

+ P.V .... [TRUNCATED]

> pcc2 <- data.frame(

+ Test = "1800mTT & 30-15IFT",

+ Estimate = cor2$estimate,

+ P.Value = cor2$p.value,

+ DF = cor3$parameter,

+ .... [TRUNCATED]

> pcc3 <- data.frame(

+ Test = "6minDT & 30-15IFT",

+ Estimate = cor3$estimate,

+ P.Value = cor3$p.value,

+ DF = cor3$parameter,

+ .... [TRUNCATED]

> # combine results into df

> combined_pccs <- rbind(pcc1, pcc2, pcc3)

> # add "± 95% CL" column

> combined_pccs$`± 95% CL` <-

+ (combined_pccs$Conf.High - combined_pccs$Conf.Low) / 2

> # round all numeric values to 2 decimals

> combined_pccs <- combined_pccs %>%

+ mutate_if(is.numeric, round, 2)

> # view combined_pccs df

> View(combined_pccs)

> # export to .xlsx

> write_xlsx(

+ list(

+ "Participant Characteristics" = parcharacteristics,

+ "Position Counts" = position_cou .... [TRUNCATED]

> # correlation label plot annotation function ----

> cor_label <- function(cor_test) {

+ bquote(italic(r) == .(round(cor_test$estimate, 2)) * .... [TRUNCATED]

> cor1_label <- cor_label(cor1)

> cor2_label <- cor_label(cor2)

> cor3_label <- cor_label(cor3)

> # linear model plots with correlation annotations

> # 1800mTT & 6minDT

> plot2 <-

+ ggplot(

+ data2,

+ aes(x = TT18_mas_m_s, y .... [TRUNCATED]

> # 1800mTT & 30-15IFT

> plot3 <-

+ ggplot(

+ data2,

+ aes(x = TT18_mas_m_s, y = ift_mas_m_s, linetype = "Linear prediction with \ ..." ... [TRUNCATED]

> # 6minDT & 30-15IFT

> plot4 <-

+ ggplot(

+ data2,

+ aes(x = DT6_mas_m_s, y = ift_mas_m_s, linetype = "Linear prediction with \n9 ..." ... [TRUNCATED]

> # combine into multi panel plot

> combined_plot <- plot2 + plot3 + plot4 + plot_layout(guides = "collect") +

+ plot_annotation(tag_levels = .... [TRUNCATED]

> # print plot

> combined_plot

> # export plot

> ggsave(

+ "Linear Models with Corr.png",

+ plot = combined_plot,

+ bg = 'white',

+ dpi = 600,

+ width = 23,

.... [TRUNCATED]

> # linear regression ------------------------------------------------------------

>

> # MAS linear model ----

> lm1 <- lm(MAS ~ Test, data = dat .... [TRUNCATED]

> summary(lm1)

Call:

lm(formula = MAS ~ Test, data = data_long)

Residuals:

Min 1Q Median 3Q Max

-0.61588 -0.20456 0.04579 0.17370 0.39090

Coefficients:

Estimate Std. Error t value Pr(>|t|)

(Intercept) 4.38582 0.04658 94.148 < 2e-16 ***

Testift_mas_m_s 0.55905 0.06588 8.486 1.42e-12 ***

TestTT18_mas_m_s 0.10394 0.06588 1.578 0.119

---

Signif. codes: 0 ‘***’ 0.001 ‘**’ 0.01 ‘*’ 0.05 ‘.’ 0.1 ‘ ’ 1

Residual standard error: 0.2375 on 75 degrees of freedom

Multiple R-squared: 0.5207, Adjusted R-squared: 0.5079

F-statistic: 40.74 on 2 and 75 DF, p-value: 1.053e-12

> # check normality of model

> check_model(lm1)

> check_heteroscedasticity(lm1)

OK: Error variance appears to be homoscedastic (p = 0.510).

> # export plot

> ggsave(

+ "Model Normality.png",

+ bg = 'white',

+ dpi = 600,

+ width = 12,

+ height = 10,

+ units = "in ..." ... [TRUNCATED]

> # extract statistics and convert to df

> lm1_summary <- tidy(lm1)

> # round all numeric values to 2 decimals

> lm1_summary <-

+ lm1_summary %>% mutate(across(where(is.numeric), round, 2))

> # view lm1_summary df

> View(lm1_summary)

> # MAS linear mixed-effects model ----

> lmm1 <- lmer(MAS ~ 1 + Test + (1 | player_id), data = data_long)

> lmm1_summary <- summary(lmm1)

> # extract statistics into df and round to 2 decimals

> lmm1_df <- data.frame(

+ Term = rownames(lmm1_summary$coefficients),

+ Estimate = .... [TRUNCATED]

> # view lmm1_df

> View(lmm1_df)

> # MAS robust repeated measures ANOVA ----

> rlmm1 <- rlmer(MAS ~ 1 + Test + (1 | player_id), data = data_long)

> rlmm1_summary <- summary(rlmm1)

> # extract statistics into df and round to 2 decimals

> rlmm1_df <- data.frame(

+ Term = rownames(rlmm1_summary$coefficients),

+ Estimate .... [TRUNCATED]

> # view rlmm1_df

> View(rlmm1_df)

> # MAS estimated marginal means ----

> emm <- emmeans(rlmm1, pairwise ~ Test, level = 0.95)

> emm_summary <-

+ summary(emm, infer = c(TRUE, TRUE)) # include p-values

> # extract statistics into df's

> emm_means <- as.data.frame(emm_summary$emmeans)

> emm_contrasts <- as.data.frame(emm_summary$contrasts)

> # combine results into a single df

> combined_emm <- bind_rows(emm_means %>% mutate(Type = "Means"),

+ emm_contrasts .... [TRUNCATED]

> # calculate new column "± 95% CL"

> combined_emm$`± 95% CL` <-

+ (combined_emm$asymp.UCL - combined_emm$asymp.LCL) / 2

> # reorder columns to position "± 95% CL" after asymp.UCL column

> combined_emm <- combined_emm %>%

+ dplyr::relocate(`± 95% CL`, .after = as .... [TRUNCATED]

> # format p-value function

> format_p_value <- function(p) {

+ if (is.na(p)) {

+ return(NA)

+ } else if (p < 0.0001) {

+ retu .... [TRUNCATED]

> # format p-values using custom function so they appear the same as in console

> combined_emm$p.value <- sapply(combined_emm$p.value, format_p_va .... [TRUNCATED]

> # round all other numeric values to 2 decimals

> combined_emm <- combined_emm %>%

+ dplyr::mutate(across(where(is.numeric) &

+ .... [TRUNCATED]

> # view combined_emm df

> print(combined_emm)

Test emmean SE df asymp.LCL asymp.UCL ± 95% CL z.ratio p.value Type contrast estimate

1 DT6_mas_m_s 4.39 0.05 Inf 4.30 4.48 0.09 95.45 <.0001 Means <NA> NA

2 ift_mas_m_s 4.96 0.05 Inf 4.87 5.05 0.09 107.94 <.0001 Means <NA> NA

3 TT18_mas_m_s 4.51 0.05 Inf 4.42 4.60 0.09 98.25 <.0001 Means <NA> NA

4 <NA> NA 0.03 Inf -0.65 -0.50 0.08 -17.50 <.0001 Contrasts DT6_mas_m_s - ift_mas_m_s -0.57

5 <NA> NA 0.03 Inf -0.21 -0.05 0.08 -3.93 0.0002 Contrasts DT6_mas_m_s - TT18_mas_m_s -0.13

6 <NA> NA 0.03 Inf 0.37 0.52 0.08 13.57 <.0001 Contrasts ift_mas_m_s - TT18_mas_m_s 0.45

> View(combined_emm)

> # export to .xlsx

> write_xlsx(

+ list(

+ "Participant Characteristics" = parcharacteristics,

+ "Position Counts" = position_cou .... [TRUNCATED]

> # HR robust repeated measures ANOVA and estimated marginal means ----

> # organise HR data long ----

> hrdata <- data %>%

+ select(

+ .... [TRUNCATED]

> hrdata_long <- pivot_longer(

+ hrdata,

+ cols = c(

+ TT18_avg_heart_rate_b_min,

+ TT18_peak_heart_rate_b_min,

+ DT6_avg_ .... [TRUNCATED]

> # view hrdata_long df

> View(hrdata_long)

> # HR robust repeated measures ANOVA ----

> rlmm2 <- rlmer(HR ~ 1 + Test + (1 | player_id), data = hrdata_long)

> rlmm2_summary <- summary(rlmm2)

> # extract statistics into df and round to 2 decimals

> rlmm2_df <- data.frame(

+ Term = rownames(rlmm2_summary$coefficients),

+ Estimate .... [TRUNCATED]

> # view rlmm2_df

> View(rlmm2_df)

> # HR estimated marginal means ----

> hremm <- emmeans(rlmm2, pairwise ~ Test, level = 0.95)

> hremm_summary <-

+ summary(hremm, infer = c(TRUE, TRUE)) # include p-values

> # extract statistics into df's

> hremm_means <- as.data.frame(hremm_summary$emmeans)

> hremm_contrasts <- as.data.frame(hremm_summary$contrasts)

> hremm_contrasts <- hremm_contrasts %>%

+ slice(c(2, 5))

> # combine results into a single df

> combined_hremm <- bind_rows(hremm_means %>% mutate(Type = "Means"),

+ hremm_c .... [TRUNCATED]

> # calculate new column "± 95% CL"

> combined_hremm$`± 95% CL` <-

+ (combined_hremm$asymp.UCL - combined_hremm$asymp.LCL) / 2

> # reorder columns to position "± 95% CL" after asymp.UCL column

> combined_hremm <- combined_hremm %>%

+ dplyr::relocate(`± 95% CL`, .after .... [TRUNCATED]

> # format p-values using custom function so they appear the same as in console

> combined_hremm$p.value <- sapply(combined_hremm$p.value, format_ .... [TRUNCATED]

> # round all other numeric values to 2 decimals

> combined_hremm <- combined_hremm %>%

+ dplyr::mutate(across(where(is.numeric) &

+ .... [TRUNCATED]

> # view combined_hremm df

> print(combined_hremm)

Test emmean SE df asymp.LCL asymp.UCL ± 95% CL z.ratio p.value Type contrast estimate

1 DT6_avg_heart_rate_b_min 176 2 Inf 172 180 4 88 <.0001 Means <NA> NA

2 DT6_peak_heart_rate_b_min 191 2 Inf 187 195 4 95 <.0001 Means <NA> NA

3 TT18_avg_heart_rate_b_min 180 2 Inf 176 184 4 90 <.0001 Means <NA> NA

4 TT18_peak_heart_rate_b_min 194 2 Inf 190 198 4 97 <.0001 Means <NA> NA

5 <NA> NA 2 Inf -9 1 5 -2 0.24 Contrasts DT6_avg_heart_rate_b_min - TT18_avg_heart_rate_b_min -4

6 <NA> NA 2 Inf -8 2 5 -2 0.42 Contrasts DT6_peak_heart_rate_b_min - TT18_peak_heart_rate_b_min -3

> View(combined_hremm)

> # export to .xlsx

> write_xlsx(

+ list(

+ "Participant Characteristics" = parcharacteristics,

+ "Position Counts" = position_cou .... [TRUNCATED]

> # percentage correspondence ----------------------------------------------------

>

> # select relevant columns and convert m_s to km_hr

> relev .... [TRUNCATED]

> # calculate percentage correspondence using raw ratio of observed data

> percentages_dt6 <- relevant_data$DT6_km_hr / relevant_data$v_ift_km_hr .... [TRUNCATED]

> percentages_tt18 <- relevant_data$TT18_km_hr / relevant_data$v_ift_km_hr * 100

> # calculate mean and SD for DT6_km_hr

> mean_percentage_dt6 <- round(mean(percentages_dt6, na.rm = TRUE), 0)

> sd_percentage_dt6 <- round(sd(percentages_dt6, na.rm = TRUE), 0)

> # calculate mean and SD for TT18_km_hr

> mean_percentage_tt18 <- round(mean(percentages_tt18, na.rm = TRUE), 0)

> sd_percentage_tt18 <- round(sd(percentages_tt18, na.rm = TRUE), 0)

> # combine results into df

> percent_correspondence <- data.frame(

+ Metric = c("DT6_km_hr", "TT18_km_hr"),

+ Mean_Percentage = c(mean_pe .... [TRUNCATED]

> # view percent_correspondence df

> print(percent_correspondence)

Metric Mean_Percentage SD_Percentage

1 DT6_km_hr 77 3

2 TT18_km_hr 79 3

> View(percent_correspondence)

> # export to .xlsx

> write_xlsx(

+ list(

+ "Participant Characteristics" = parcharacteristics,

+ "Position Counts" = position_cou .... [TRUNCATED]

> # regression equations for estimating 6minDT and 1800mTT MAS from vIFT ---------

>

> # robust model for DT6_mas_m_s and vIFT

> model_dt6 <- lmr .... [TRUNCATED]

> percentages_dt6 <- (coef(model_dt6)["v_ift_km_hr"] * relevant_data$v_ift_km_hr) / relevant_data$DT6_km_hr * 100

> mean_percentage_dt6 <- round(mean(percentages_dt6, na.rm = TRUE), 0)

> sd_percentage_dt6 <- round(sd(percentages_dt6, na.rm = TRUE), 0)

> # robust model for TT18_mas_m_s and vIFT

> model_tt18 <- lmrob(TT18_km_hr ~ v_ift_km_hr, data = relevant_data)

> percentages_tt18 <- (coef(model_tt18)["v_ift_km_hr"] * relevant_data$v_ift_km_hr) / relevant_data$TT18_km_hr * 100

> mean_percentage_tt18 <- round(mean(percentages_tt18, na.rm = TRUE), 0)

> sd_percentage_tt18 <- round(sd(percentages_tt18, na.rm = TRUE), 0)

> # view models

> summary(model_dt6)

Call:

lmrob(formula = DT6_km_hr ~ v_ift_km_hr, data = relevant_data)

\--> method = "MM"

Residuals:

Min 1Q Median 3Q Max

-1.307065 -0.465018 -0.002212 0.434420 1.704811

Coefficients:

Estimate Std. Error t value Pr(>|t|)

(Intercept) 3.2409 3.2372 1.001 0.32675

v_ift_km_hr 0.6125 0.1543 3.969 0.00057 ***

---

Signif. codes: 0 ‘***’ 0.001 ‘**’ 0.01 ‘*’ 0.05 ‘.’ 0.1 ‘ ’ 1

Robust residual standard error: 0.6865

Multiple R-squared: 0.4198, Adjusted R-squared: 0.3956

Convergence in 12 IRWLS iterations

Robustness weights:

2 weights are ~= 1. The remaining 24 ones are summarized as

Min. 1st Qu. Median Mean 3rd Qu. Max.

0.5170 0.9105 0.9596 0.9223 0.9810 0.9969

Algorithmic parameters:

tuning.chi bb tuning.psi refine.tol rel.tol scale.tol solve.tol zero.tol eps.outlier eps.x warn.limit.reject warn.limit.meanrw

1.548e+00 5.000e-01 4.685e+00 1.000e-07 1.000e-07 1.000e-10 1.000e-07 1.000e-10 3.846e-03 4.002e-11 5.000e-01 5.000e-01

nResample max.it best.r.s k.fast.s k.max maxit.scale trace.lev mts compute.rd fast.s.large.n

500 50 2 1 200 200 0 1000 0 2000

psi subsampling cov compute.outlier.stats

"bisquare" "nonsingular" ".vcov.avar1" "SM"

seed : int(0)

> summary(model_tt18)

Call:

lmrob(formula = TT18_km_hr ~ v_ift_km_hr, data = relevant_data)

\--> method = "MM"

Residuals:

Min 1Q Median 3Q Max

-2.26881 -0.46439 0.03882 0.25385 1.05721

Coefficients:

Estimate Std. Error t value Pr(>|t|)

(Intercept) -0.9326 3.7460 -0.249 0.806

v_ift_km_hr 0.8418 0.1758 4.789 7.1e-05 ***

---

Signif. codes: 0 ‘***’ 0.001 ‘**’ 0.01 ‘*’ 0.05 ‘.’ 0.1 ‘ ’ 1

Robust residual standard error: 0.3675

Multiple R-squared: 0.7095, Adjusted R-squared: 0.6974

Convergence in 31 IRWLS iterations

Robustness weights:

observation 7 is an outlier with |weight| = 0 ( < 0.0038);

one weight is ~= 1. The remaining 24 ones are summarized as

Min. 1st Qu. Median Mean 3rd Qu. Max.

0.0980 0.7759 0.9180 0.8147 0.9788 0.9959

Algorithmic parameters:

tuning.chi bb tuning.psi refine.tol rel.tol scale.tol solve.tol zero.tol eps.outlier eps.x warn.limit.reject warn.limit.meanrw

1.548e+00 5.000e-01 4.685e+00 1.000e-07 1.000e-07 1.000e-10 1.000e-07 1.000e-10 3.846e-03 4.002e-11 5.000e-01 5.000e-01

nResample max.it best.r.s k.fast.s k.max maxit.scale trace.lev mts compute.rd fast.s.large.n

500 50 2 1 200 200 0 1000 0 2000

psi subsampling cov compute.outlier.stats

"bisquare" "nonsingular" ".vcov.avar1" "SM"

seed : int(0)

> # create regression equation + residual standard error function

> generate_equation <- function(model) {

+ coefficients <- coef(model)

+ .... [TRUNCATED]

> # generate equations and residual standard errors for 6minDT & 1800mTT

> result1 <- generate_equation(model_dt6)

> result2 <- generate_equation(model_tt18)

> equation1 <- result1$equation

> equation2 <- result2$equation

> se1 <- result1$residual_se

> se2 <- result2$residual_se

> # create df with equations and residual standard errors

> equations_df <- data.frame(

+ Model = c("Model 1", "Model 2"),

+ Equation = c( .... [TRUNCATED]

> # view equations_df

> print(equations_df)

Model Equation Standard.Error

1 Model 1 DT6_km_hr = 3.24 + 0.61*v_ift_km_hr 0.69

2 Model 2 TT18_km_hr = -0.93 + 0.84*v_ift_km_hr 0.37

> View(equations_df)

> # export to .xlsx

> write_xlsx(

+ list(

+ "Participant Characteristics" = parcharacteristics,

+ "Position Counts" = position_cou .... [TRUNCATED]

> # cite R, packages used, RStudio and environment -------------------------------

>

> # R, packages & package versions

> cite_packages(cite.tidy .... [TRUNCATED]

|

| | 0%

|

|..................................................................................................................................................................................................................| 100%

/Applications/RStudio.app/Contents/Resources/app/quarto/bin/tools/aarch64/pandoc +RTS -K512m -RTS grateful-report.knit.md --to docx --from markdown+autolink_bare_uris+tex_math_single_backslash --output /Users/kieransmith/Desktop/grateful-report.docx --lua-filter /Library/Frameworks/R.framework/Versions/4.4-arm64/Resources/library/rmarkdown/rmarkdown/lua/pagebreak.lua --highlight-style tango --citeproc

[1] "./grateful-report.docx"

> # RStudio

> RStudio.Version()

$citation

To cite RStudio in publications use:

Posit team (2025). RStudio: Integrated Development Environment for R. Posit Software, PBC, Boston, MA. URL http://www.posit.co/.

A BibTeX entry for LaTeX users is

@Manual{,

title = {RStudio: Integrated Development Environment for R},

author = {{Posit team}},

organization = {Posit Software, PBC},

address = {Boston, MA},

year = {2025},

url = {http://www.posit.co/},

}

$mode

[1] "desktop"

$version

[1] ‘2024.12.1.563’

$long_version

[1] "2024.12.1+563"

$release_name

[1] "Kousa Dogwood"

> # environment

> sessionInfo()

R version 4.4.3 (2025-02-28)

Platform: aarch64-apple-darwin20

Running under: macOS Sequoia 15.3.2

Matrix products: default

BLAS: /System/Library/Frameworks/Accelerate.framework/Versions/A/Frameworks/vecLib.framework/Versions/A/libBLAS.dylib

LAPACK: /Library/Frameworks/R.framework/Versions/4.4-arm64/Resources/lib/libRlapack.dylib; LAPACK version 3.12.0

locale:

[1] en_US.UTF-8/en_US.UTF-8/en_US.UTF-8/C/en_US.UTF-8/en_US.UTF-8

time zone: Europe/London

tzcode source: internal

attached base packages:

[1] stats graphics grDevices utils datasets methods base

other attached packages:

[1] grateful_0.2.11 robustbase_0.99-4-1 emmeans_1.11.0 robustlmm_3.3-1 lme4_1.1-36 Matrix_1.7-3 performance_0.13.0 broom_1.0.7 patchwork_1.3.0 writexl_1.5.2

[11] lubridate_1.9.4 forcats_1.0.0 stringr_1.5.1 dplyr_1.1.4 purrr_1.0.4 readr_2.1.5 tidyr_1.3.1 tibble_3.2.1 ggplot2_3.5.1 tidyverse_2.0.0

[21] janitor_2.2.1 readxl_1.4.5

loaded via a namespace (and not attached):

[1] tidyselect_1.2.1 farver_2.1.2 fastmap_1.2.0 bayestestR_0.15.2 digest_0.6.37 timechange_0.3.0 estimability_1.5.1 lifecycle_1.0.4 fastGHQuad_1.0.1 magrittr_2.0.3 compiler_4.4.3

[12] rlang_1.1.5 tools_4.4.3 yaml_2.3.10 knitr_1.50 labeling_0.4.3 rsconnect_1.3.4 withr_3.0.2 grid_4.4.3 datawizard_1.0.1 xtable_1.8-4 colorspace_2.1-1

[23] scales_1.3.0 MASS_7.3-65 insight_1.1.0 cli_3.6.4 mvtnorm_1.3-3 rmarkdown_2.29 crayon_1.5.3 ragg_1.3.3 reformulas_0.4.0 generics_0.1.3 rstudioapi_0.17.1

[34] tzdb_0.5.0 minqa_1.2.8 splines_4.4.3 cellranger_1.1.0 vctrs_0.6.5 boot_1.3-31 hms_1.1.3 ggrepel_0.9.6 systemfonts_1.2.1 see_0.11.0 glue_1.8.0

[45] nloptr_2.2.1 DEoptimR_1.1-3-1 codetools_0.2-20 stringi_1.8.4 gtable_0.3.6 munsell_0.5.1 pillar_1.10.1 htmltools_0.5.8.1 R6_2.6.1 textshaping_1.0.0 Rdpack_2.6.3

[56] evaluate_1.0.3 lattice_0.22-6 rbibutils_2.3 backports_1.5.0 snakecase_0.11.1 renv_1.1.4 Rcpp_1.0.14 coda_0.19-4.1 nlme_3.1-167 xfun_0.51 mgcv_1.9-1

[67] pkgconfig_2.0.3

> # TO DO ------------------------------------------------------------------------

>

> #

>

>

>

>
